# The assessment of readiness to change among head managers of primary healthcare centers in Makkah, KSA

**DOI:** 10.1016/j.jtumed.2024.02.005

**Published:** 2024-03-08

**Authors:** Turky J. Arbaein, Khulud K. Alharbi, Abdulrhman A. Alzhrani, Sarah S. Monshi, Ali M. Alzahrani, Talal M. Alsadi

**Affiliations:** a Department of Health Administration and Hospital, College of Public Health and Health Informatics, Umm Al-Qura University, Makkah, KSA; b Al Awali Primary Healthcare Department, Makkah Healthcare Cluster, KSA

**Keywords:** إدارة التغيير, الجاهزية للتغيير, التحول الصحي السعودي, نموذج أدكار, الرعاية الصحية الأولية, ADKAR model, Change management, Change readiness, Primary healthcare center, Saudi health transformation

## Abstract

**Background:**

KSA is currently undergoing significant changes in its healthcare system, with a particular emphasis on enhancing the role of primary healthcare centers (PHCs) to elevate patient experience and overall healthcare quality. At the forefront of this transformation are head managers in PHCs, who play a crucial role in implementing these changes effectively. The readiness of these managers is paramount to the successful execution of the envisioned transformation and the subsequent improvement of patient experience.

**Objective:**

This study aims to assess the readiness to change among head managers of primary healthcare centers in Makkah, KSA.

**Methodology:**

Cross-sectional study utilized the ADKAR model questionnaire, consisting of 22 Likert scale questions, to assess PHCs head managers' awareness, desire, knowledge, ability, reinforcement, and overall change readiness.

**Results:**

The study found a significant association between higher educational levels and increased awareness (β = 0.214, p = 0.030), along with greater desire (β = 0.207, p = 0.029) among primary healthcare (PHC) managers. Additionally, a positive association was found between age (≥41 years) and knowledge among PHC managers (β = 0.138, p = 0.030). However, managers with 11 or more years of experience showed a negative association with change readiness (β = −0.112, p = 0.001).

**Conclusion:**

The ADKAR model outlines five dimensions that are useful for identifying the readiness and willingness of head managers in PHCs in Makkah cluster to undergo change. Assessing change readiness is crucial for organizational transformation, with head managers playing a significant role. Factors such as age, education, and experience influence managers' readiness for change in primary healthcare centers (PHCs) in Makkah.

## الملخص

**أهداف البحث:**

تشهد المملكة العربية السعودية تغييرات تحويلية في مجال الرعاية الصحية، مع إعطاء الأولوية لمراكز الرعاية الصحية الأولية لتحسين تجربة المرضى ونظام الرعاية الصحية الشامل. وتهدف هذه الدراسة إلى تقييم مدى الاستعداد للتغيير لدى مديري مراكز الرعاية الصحية الأولية في مكة المكرمة، المملكة العربية السعودية.

**طرق البحث:**

استخدمت دراسة مقطعية نموذج استبانة "أدكار"، التي تتكون من 22 سؤالاً على مقياس ليكرت، لتقييم وعي المديرين ورغبتهم ومعرفتهم وقدراتهم وتعزيزهم واستعدادهم للتغيير بشكل عام.

**النتائج:**

وجدت الدراسة وجود ارتباط كبير بين مستويات التعليم العالي وزيادة الوعي، إلى جانب زيادة الرغبة، بين مديري الرعاية الصحية الأولية. بالإضافة إلى ذلك، تم العثور على ارتباط إيجابي بين العمر (أكبر من 41 سنة) والمعرفة بين مديري الرعاية الصحية الأولية. ومع ذلك، أظهر المديرون الذين لديهم 11 عامًا أو أكثر من الخبرة ارتباطا سلبيا بالاستعداد للتغيير.

**الاستنتاجات:**

يحدد نموذج "أدكار" خمسة أبعاد مفيدة لتحديد مدى استعداد المديرين واستعدادهم للخضوع للتغيير. تعتمد إدارة التغيير الناجحة على انفتاح الأفراد على التغيير، متأثرين بعوامل مثل العمر والتعليم والخبرة.

## Introduction

KSA is undergoing significant transformative changes in its healthcare structure and system. Numerous transformative healthcare programs were launched in 2021 as part of the Kingdom Vision 2030, to restructure the health sector. The primary objective of these programs is to establish a comprehensive, effective, and integrated health system.^1^ The Kingdom has prioritized primary healthcare centers (PHC) in its newly proposed Model of Care.^2^ PHC is a crucial component of an effective healthcare system, serving as the first line of interaction between the patient and the healthcare system.^2^ Consequently, executive directors in the Ministry of Health have initiated several projects to enhance the patient experience and the contribution of PHCs in the Saudi Arabian healthcare system. Various projects, including health promotion campaigns, medical consultations via social media platforms, and comprehensive frontline employee initiatives, have been announced to achieve these goals.^3^ These projects aim to shift the nature of employees' tasks in PHC from single-task-oriented to a more versatile, multiple-task orientation.^4^ This substantial change requires change management as well as preparedness before the implementation phase. The lack of effective change management and preparedness could lead to a change-resistant attitude, a decline in job satisfaction rates, or an increase in turnover rates among PHC employees.^2^

Change management plays an increasingly important role in modern organizations in different industries including healthcare.^5^ Change readiness is a fundamental component of change management, which is the structured approach to transitioning individuals, teams, and organizations from their current state to a desired future state.^6^ Change readiness specifically focuses on assessing the willingness and ability of individuals within an organization to embrace and adapt to change.^7^ There is strong evidence that when readiness in an organization is high, the organization is better able to initiate and sustain a major change.^8^ Thus, assessing change readiness is pivotal, particularly in determining the level of readiness for effective change management practices.

Measuring change readiness can help organizations identify potential barriers to change and develop strategies to address them. Several tools are available to measure change readiness, including surveys, interviews, focus groups, and observation.^9^ One widely recognized framework for measuring change readiness effectively is Prosci's ADKAR model.^10^ The ADKAR model is a useful tool for identifying the readiness and willingness of individuals or organizations to undergo a change. The model outlines five dimensions that an individual must achieve to be ready for a desired change successfully: 1- Awareness: determine if individuals understand the need for the change and its potential impact. 2- Desire: assess if individuals are willing to undergo the change. 3- Knowledge: determine if individuals have the necessary skills and knowledge to undergo the change. 4- Ability: evaluate if individuals have the resources and support necessary to implement the change. 5- Reinforcement: determine if individuals have the motivation and support necessary to sustain the change over time.^11^

The Prosci's ADKAR model has been widely employed across diverse industries such as education and information technology to evaluate and facilitate the implementation of new processes, technologies, and initiatives.^12^^,^^13^ For instance, Adel et al. employed the ADKAR model to investigate barriers to change management in the public sector of educational institutions. They identified both strengths and weaknesses in their analysis. The study found that the ‘desire’ dimension exhibited strength, indicating a strong motivation for change. However, weaknesses were observed in the ‘knowledge’ dimension, particularly due to the absence of predetermined guidelines.^12^ Also, Silvia utilizes the ADKAR change management model to explore the intervention needed to enhance accessibility in web portals. The results revealed high awareness of the need for change but indicate a need for intervention in reinforcement to sustain change over the long term.^13^

Similarly, in healthcare settings where changes often involve complex systems and processes, the ADKAR model has demonstrated its applicability and effectiveness by breaking down change into manageable components.^14^^,^^15^ This model helps healthcare organizations identify areas where individuals or groups may face resistance or challenges. For example, Nestor and Gideon conducted a study to assess nursing perceptions and readiness to combat gender-based violence (GBV) during the COVID-19 pandemic in Namibia using the ADKAR model.^14^ The results indicated that the readiness was highest in the reinforcement and awareness dimensions with gender and age were significant predictors of readiness.^14^ Moreover, Alice et al. investigated nurses' readiness to use nursing Kardex by applying the ADKAR Model in hospitals.^15^ They determined the need to enhance nurses in two dimensions: knowledge and ability to use nursing Kardex, particularly in non-educational hospitals.^15^

To successfully implement the transformative projects in primary healthcare (PHC) in KSA, particularly in Makkah city, it is essential for every PHC to undergo change, and the success of this change depends on the employees. Among these employees, PHC managers play crucial roles in visioning, enlisting, empowering, monitoring, and helping other employees adopt the change.^9^ Managers must be equipped with the necessary skills and knowledge to navigate changes, communicate effectively with stakeholders, and establish a culture that embraces change.^9^ When managers are not ready for change, they may resist or undermine efforts to implement new policies or practices, leading to failure in achieving the desired outcomes.^9^

In this study, Prosci's ADKAR model has been recognized as effectively meeting the specific needs of primary healthcare in Makkah. The framework offered by the model enables us to assess the level of preparedness for the changes that aim to transform Makkah PHC managers from a conventional-task to a transformative-task orientation. Additionally, the model helps identify any potential obstacles or resistance to the desired change. Therefore, ensuring the readiness of PHC managers for the projects aimed at improving the patient experience in PHC in Makkah city can be seen as a major topic in adopting transformative change in healthcare organizations.

While previous studies have acknowledged the importance of management in driving change, there is limited research focusing on evaluating the readiness of PHC managers in this context. This study aims to assess the readiness to change among managers of primary healthcare centers in Makkah, KSA.

## Materials and Methods

### Study design

We conducted a cross-sectional study involving the heads managers from 82 PHCs in Makkah healthcare cluster. Our primary objective was to assess the preparedness of PHCs head managers for transformative change. Data collection was facilitated through the utilization of the ADKAR model questionnaire.^16^

### Study population and sample size

The target population of this study was 82 heads managers of the 82 PHCs in the Makkah cluster. A purposive sampling method was employed, using the Raosoft sample size calculator (http://www.raosoft.com/samplesize.html) to determine the sample size. With a standard deviation of 1.96 for a 99% confidence interval, a 5% margin of error, an anticipated 50% response rate, and a target population of 82 managers across PHC in the Makkah cluster, the minimum required sample size was calculated to be 74.

### Data collection

Data collection took place between January and February 2023. The survey was distributed through a popular social media platform, WhatsApp. An authorized member of the Makkah cluster coordinated the distribution of the survey, directly reaching and inviting the intended participants anonymously. This approach significantly increased participation, resulting in 76 respondents out of the 82-target population. Participants' completion and submission of the survey were considered as consent for their data to be included in the study. All the participants were fully aware of the purpose of this questionnaire and reasons for collecting the data. The researchers ensured the security of any information collected from participants and its use solely for intended research purposes. Access to the data was restricted to only authorised researchers.

### Study instrument

The ADKAR model is a Prosci Methodology model that researchers have successfully applied in various fields, such as education,^17^ business,^18^ information technology,^13^ and healthcare.^14^ We selected ADKAR model for its reliability, validity, and alignment with our study aim. We measured the 5 dimensions of the ADKAR model using 22 questions that participants answered on a 5-point Likert scale from 1 (strongly disagree) to 5 (strongly agree).

The ADKAR framework comprises five dimensions, each with four to five questions, to assess managers' readiness for change. These dimensions include Awareness of the need for change, Desire to participate in and support the change, Knowledge required for the change, Ability to implement necessary behavior changes, and Reinforcement to sustain the change. Additionally, we created a total score of the five dimensions, and we called it a Change Readiness. To ensure the reliability of the survey, Cronbach's alpha (α) was calculated for each dimension: Awareness (α = 0.73), Desire (α = 0.84), Knowledge (α = 0.9192), Ability (α = 0.85), and Reinforcement (α = 0.87). The overall reliability for the survey-change readiness-was α = 0.95.

### Outcome variables

The outcome variables in this study included five variables that corresponded to the five dimensions of the ADKAR model, which are managers' Awareness, Desire, Knowledge, Ability, and Reinforcement along with a sixth variable representing the managers' change readiness. To construct each of the five variables, the sum score of the questions associated with each dimension was utilized. Similarly, the managers’ change readiness was calculated by adding up the sum score of all the dimensions.

### Independent variables

The study included four binary and categorical variables: age, gender, education, and experience. To ensure that we had sufficient observations in each category, we combined the age groups of 20–30 and 31–40 into one category and 41–50 and 51–60 into another category. We also combined those with Master's and PhD degrees into one group due to the small sample size, while high school and undergraduate degrees were left as separate categories. Finally, we combined those with work experience from 4 to 7 and 8–11 years into one group, leaving those with 1–3 years of experience and more than 11 years of experience as separate groups.

### Data Analysis

Descriptive statistics were reported for the study sample. We conducted bivariate analyses using one-way ANOVA to examine whether there were significant differences in the dimension scores' means among various levels of the independent variables. To identify individual-level factors associated with readiness to change, several Poisson models were fitted and analyzed - one for each dimension as well as the total score. The analyses were performed using Stata (17, StataCorp LLC, College Station, TX).

## Results

A total of 76 managers of the PHCs in Makkah completed and returned the questionnaire, resulting in a 95% response rate. The majority of participating managers were males (78.95%), aged 20–40 years old (61.84%), had completed an undergraduate degree (62.16%), and had ≥11 years of experience in practice (44.74%). Table 1 displays the demographic characteristics of the study sample.

**Table 1:** Demographic characteristics of the study sample.

Individual characteristics	N	%
**Age groups (years)**		
20–40	47	61.84
≥41	29	38.16
**Gender**		
Female	16	21.05
Male	60	78.95
**Education**		
High School	18	24.32
Undergraduate degree	46	62.16
Graduate degree	10	13.51
**Experience (years)**		
1–3	21	27.63
4–11	21	27.63
≥11	34	44.74

Table 2 shows the mean and standard deviation of dimension scores for each level of the study independent variables. It also reports the results of bivariate analyses for examining differences in the mean dimension scores among levels of the independent variables. The bivariate analyses revealed no statistically significant differences among groups across most survey dimensions, with the exception of awareness. Specifically, the analysis revealed significant differences in awareness based on education levels (P < 0.05). Participants holding Master's degrees demonstrated a higher awareness of change readiness compared to other groups [Mean (M): 24, Standard deviation (SD): 1].

**Table 2:** Distribution of dimensions’ scores by study independent variables.

	Change readiness		Awareness		Desire		Knowledge		Ability		Reinforcement	
	Mean (SD)	*p*-value	Mean (SD)	*p*-value	Mean (SD)	*p*-value	Mean (SD)	*p*-value	Mean (SD)	*p*-value	Mean (SD)	*p*-value

Gender		0.26		0.63		0.20		0.18		0.38		0.30
Female	89.25 (13.02)		17.6 (3.18)		21 (3.30)		24.93 (5.19)		14.81 (3.10)		11.75 (2.40)	
Male	83.18 (20.50)		17.03 (4.3)		19.22 (5.23)		22.59 (6.46)		13.82 (4.21)		10.83 (3.30)	
Age		0.91		0.61		0.94		0.96		0.52		0.34
20–30	87.60 (18.55)		16.2 (5.35)		20.8 (7.25)		24 (6.40)		14.8 (1.30)		11.8 (1.64)	
31–40	83.47 (15.10)		17.73 (3.40)		19.60 (3.35)		22.85 (5.32)		13.5 (3.67)		10.63 (2.97)	
41–50	84.54 (26.50)		16.54 (5.28)		19.34 (6.78)		23.12 (8.07)		14.40 (4.96)		11.08 (3.63)	
51–60	89.2 (13.36)		16.2 (4.15)		19.8 (4.60)		24 (4.74)		16 (3.08)		13.2 (2.50)	
Education		0.26		**0.00**		0.18		0.44		0.51		0.68
BA	84.23 (18.86)		16.73 (3.70)		19.60 (4.22)		23.50 (6.12)		14.09 (4.31)		10.82 (3.31)	
HS	80.22 (23.67)		16.33 (4.90)		17.88 (6.74)		21.22 (7.50)		13.50 (3.85)		11.22 (3.24)	
MA	103.66 (6.50)		24 (1)		23.66 (1.15)		26.66 (2.51)		17 (2.64)		12.33 (0.60)	
PhD	88.14 (9.26)		19.85 (0.69)		21.14 (3.23)		23 (4.6)		13.14 (1.95)		11 (3.14	
# Of working years		0.17		0.53		0.07		0.16		0.12		0.29
3 or less	89 (15.11)		16.95 (3.23)		21.05 (4.14)		24.23 (5.70)		15.52 (3.62)		11.90 (2.62)	
4 to 7	92.55 (9.36)		17.88 (3.10)		22.11 (2.36)		26.33 (3.84)		14.77 (2.27)		11.44 (1.42)	
8 to 11	84.41 (18.31)		18.5 (4.56)		19.25 (5.22)		22.81 (5.23)		13.90 (4.03)		11.27 (2.57)	
More than 11	79.52 (22.69)		16.58 (4.71)		18.18 (5.38)		21.61 (7.09)		12.90 (4.36)		10.30 (3.80)	

Significant differences in awareness based on education levels (P < 0.05) are mentioned in bold.

Table 3 presents the results of a multivariable analysis examining the association between individual characteristics and five dimensions of the ADKAR model, which are related to change readiness among healthcare managers in Makkah PHC. The table shows the regression coefficients (β) and p-values for each individual characteristic, including age, gender, education, and experience, with respect to the dimensions of awareness, desire, knowledge, ability, reinforcement, and change readiness.

**Table 3:** Individual characteristics associated with outcomes of interest, awareness, desire, knowledge, ability, reinforcement and change readiness.

Individual Characteristics	Awareness		Desire		Knowledge		Ability		Reinforcement		Change Readiness	
	β	*p*-value	β	*p*-value	β	*p*-value	β	*p*-value	β	*p*-value	β	*p*-value
**Age groups (years)**												
20–40	Ref											
≥41	−0.049	0.505	0.102	0.150	0.138	0.030∗	0.173	0.034∗	0.114	0.216	0.095	0.005∗
**Gender**												
Female	Ref											
Male	0.005	0.943	−0.073	0.305	−0.074	0.258	−0.068	0.424	−0.085	0.374	−0.062	0.071
**Education**												
High School	Ref											
Undergraduate degree	−0.016	0.844	0.107	0.159	0.136	0.049∗	0.093	0.295	−0.020	0.835	0.080	0.031∗
Graduate degree	0.214	0.030∗	0.207	0.029∗	0.146	0.099	0.092	0.421	0.018	0.885	0.161	0.001 ∗∗
**Experience (years)**												
1–3	Ref											
4–11	0.028	0.731	−0.006	0.942	0.040	0.586	−0.025	0.791	−0.021	0.847	0.006	0.867
>11	−0.023	0.741	−0.114	0.087	−0.088	0.147	−0.167	0.031∗	−0.146	0.091	−0.112	0.001∗

∗∗p < 0.001, ∗p < 0.05.

The results revealed that education was significantly associated with awareness and desire; having a graduate degree was positively associated with both awareness (β = 0.214, p = 0.030) and desire (β = 0.207, p = 0.029). Age and education were also significantly associated with knowledge; with participants aged ≥41 years old and those having an undergraduate degree showing positive association (β = 0.138, p = 0.030) and (β = 0.136, p = 0.049), respectively. Moreover, the results showed that both age and experience had a significant association with ability. Being ≥41 years old had a positive association with ability (β = 0.138, p = 0.030), while having ≥11 years of experience had a negative association with ability (β = −0.167, p = 0.031). However, the results found no significant associations between any of the individual characteristics and reinforcement.

Finally, significant associations were found between individual characteristics (age, education, and experience) and change readiness. Being ≥41 years old, having an undergraduate degree, and having a graduate degree were positively associated with change readiness (β = 0.095, p = 0.005) (β = 0.080, p = 0.031) and (β = 0.161, p < 0.001), respectively. However, having an experience of ≥11 years was negatively associated with the change readiness (β = −0.112, p = 0.001).

## Discussion

To the best of our knowledge, this research is the inaugural investigation to explore the readiness for change among PHCs head managers who are employed in the government sector of KSA. This study revealed a statistical relationship between individual characteristics. Including age, education level, experience, and change readiness among PHC managers in Makkah City. Managers with a graduate degree are positively associated with the awareness and desire to make a change in employees tasks orientation in Makkah PHC to enhance the patient experience. Compared to young managers, senior managers (aged ≥41) were more knowledgeable about the change needed and equipped with essential skills to execute the change in PHCs. In contrast, healthcare managers with higher work experience were less prepared for change.

At the individual level of managers the findings of this study indicated that age is a significant factor influencing the readiness to change. Age demonstrates how an individual can change over time and how his/her performance may change. However, research demonstrates that age alone does not determine an individual's readiness to change.^19^ Age can be a proxy for experience, skills, and knowledge, which are critical factors that impact change readiness.^19^ Thus, the experience can mitigate the effects of age, making older workers more adaptable to change.

Education level is another identified factor that impacts change readiness among PHC managers in Makkah City. Aligned with our results, studies indicate that managers with a higher level of education are more likely to have a growth mindset and be more open to learning new things.^20^ They are also more likely to have a broader perspective and can better recognize the benefits of change. It is reported that highly educated individuals have the ability to analyze and process information,^20^ making them better equipped to embrace a desired change by organization. These study findings encourage future studies to gather information about personal characteristics, organizational culture, management styles, and employees' willingness to change before initiating organizational changes. This approach may help assess the readiness to change and mitigate future change resistance.

Interestingly, the findings of this study showed a negative association between managers’ years of experience and overall change readiness, in particular, the ability to make changes within healthcare settings. Previous studies have found that employees' change readiness levels vary depending on their experience level. According to Sterlacci (2020), employees with less than ten years of experience tend to be more open to change than those with more than ten years of experience.^21^ These findings suggest that the degree of change readiness may decrease as employees gain more work experience. In addition, employees who have worked in a particular organization for an extended period tend to have established routines and practices. Such habits make them resistant to adapting to new changes.^22^ Within the context of this study, the Saudi healthcare system was previously characterized by bureaucracy and centralization, which negatively impact creativity and speed of work tasks. These system characteristics may explain the lack of change readiness among highly experienced PHC managers in Makkah City. It is promising that the new transformation of the healthcare plan includes decentralization, which would grant more freedom to managers in making decisions within the healthcare system.

The limited degree of change readiness among healthcare managers could be explained by the several challenges that the Saudi healthcare system has faced, such as shifting the paradigm from curative medicine to preventive medicine, applying the privatization strategy in the health sector, and finally overcoming Covid pandemic.^23^^,^^24^ More experienced healthcare managers had undergone so many changes during all these events that they felt “sick to the back teeth”.^21^ As a result, they may acknowledge the need to change. However, at the same time, they tend to avoid engagement in any change plan as a defensive mechanism to detach or withdraw from unpleasant feelings associated with the change.

Although the recent transformation in the healthcare sector in KSA creates the urge to make change, it may develop a sense of job insecurity and future uncertainty among experienced healthcare employees, leading to resistance.^25^ Consequently, this study emphasizes the notion of positive organizational culture supporting employees during any change initiatives. Adequate support in the form of communication, training, and education could mitigate the influences of individual-level variations resulting from age, gender, education, and experience, which would improve the readiness to change. To reduce resistance to change, upper-level healthcare managers must ensure that all employees understand the rationale behind implementing any changes and provide essential resources to execute tasks related to change initiatives.

### Limitation

This study has several limitations that should be considered. First, the sample size was relatively small, which may limit the generalizability of the findings to other populations. Second, the study focused only on primary healthcare managers in a specific region, and the findings may not be representative of managers in other regions or healthcare settings. Finally, self-reported bias may have affected the accuracy and reliability of the data collected from the questionnaire.

## Conclusion

Assessing change readiness is vital for any organization undergoing transformation. Our study highlights that the readiness of head managers plays a significant role in this process. We found that age, education, and experience are critical factors influencing managers' readiness to embrace change in PHCs within the Makkah cluster. Specifically, older and more educated head managers demonstrated higher readiness for change, while those with extensive experience showed resistance. Organizations must recognize these factors to effectively implement changes. Understanding employees' readiness levels before initiating changes is essential, and providing adequate support and encouragement during the transition is crucial. When employees feel valued and supported, they are more likely to embrace organizational changes, leading to successful outcomes.

## Source of funding

This research did not receive any specific grant from funding agencies in the public, commercial, or not-for-profit sectors.

## Conflict of interest

The authors declare that there are no conflicts of interest.

## Ethical approval

The study followed all ethical standards, and it was approved by Umm Al-Qura University Biomedical Research Ethics Committee with the reference number (HAPO-02-K-012-2023-01- 1399) on January 23, 2023.

## Author contributions

TJA conceived and designed the study, conducted research, formulated the research objective, and contributed to the writing up. TMS and AMA collected and organized data. AAA analyzed and interpreted data. SSM wrote an initial draft of article and provided logistic support. KKA contributed to the methodology part of the article. All authors have critically reviewed and approved the final draft and are responsible for the content and similarity index of the manuscript.
